# Metagenomes and metatranscriptomes from boreal potential and actual acid sulfate soil materials

**DOI:** 10.1038/s41597-019-0222-3

**Published:** 2019-10-16

**Authors:** Eva Högfors-Rönnholm, Margarita Lopez-Fernandez, Stephan Christel, Diego Brambilla, Marcel Huntemann, Alicia Clum, Brian Foster, Bryce Foster, Simon Roux, Krishnaveni Palaniappan, Neha Varghese, Supratim Mukherjee, T. B. K. Reddy, Chris Daum, Alex Copeland, I-Min A. Chen, Natalia N. Ivanova, Nikos C. Kyrpides, Miranda Harmon-Smith, Emiley A. Eloe-Fadrosh, Daniel Lundin, Sten Engblom, Mark Dopson

**Affiliations:** 1 Research and Development, Novia University of Applied Sciences, Vaasa, 65200 Finland; 2 Centre for Ecology and Evolution in Microbial Model Systems (EEMiS), Linnaeus University, Kalmar, 59231 Sweden; 3 Department of Energy Joint Genome Institute, Walnut Creek, CA 94598 USA

**Keywords:** Biogeochemistry, Sequencing, Metagenomics, Environmental impact

## Abstract

Natural sulfide rich deposits are common in coastal areas worldwide, including along the Baltic Sea coast. When artificial drainage exposes these deposits to atmospheric oxygen, iron sulfide minerals in the soils are rapidly oxidized. This process turns the potential acid sulfate soils into actual acid sulfate soils and mobilizes large quantities of acidity and leachable toxic metals that cause severe environmental problems. It is known that acidophilic microorganisms living in acid sulfate soils catalyze iron sulfide mineral oxidation. However, only a few studies regarding these communities have been published. In this study, we sampled the oxidized actual acid sulfate soil, the transition zone where oxidation is actively taking place, and the deepest un-oxidized potential acid sulfate soil. Nucleic acids were extracted and 16S rRNA gene amplicons, metagenomes, and metatranscriptomes generated to gain a detailed insight into the communities and their activities. The project will be of great use to microbiologists, environmental biologists, geochemists, and geologists as there is hydrological and geochemical monitoring from the site stretching back for many years.

## Abstract (summary)

**Table:** 

Measurement(s)	rRNA_16S • Metagenome • transcription profiling assay
Technology Type(s)	amplicon sequencing • DNA sequencing • RNA sequencing
Factor Type(s)	biological replicate • sampling depth
Sample Characteristic - Environment	soil biocrust
Sample Characteristic - Location	Finland

Machine-accessible metadata file describing the reported data: 10.6084/m9.figshare.9901265

## Background & Summary

Naturally occurring deposits containing sulfidic sediments that form acid sulfate soils (ASS) cover over 17 million hectares of coastal areas. These extreme soils occur in North America (e.g. in the South and South West of the U.S) as well as in Europe (e.g. surrounding the Baltic Sea), Asia, and Australia^1^. Exposure of the sulfidic materials to air initializes chemical reactions that produce sulfuric acid^2^, thus creating ASS with a pH < 4, while mobilizing large quantities of acidity and leachable toxic metal(loid)s (e.g. Al, As, Cd, Co, Ni, and Zn). The leached metals and acidity are ultimately transported to the surrounding waters where they cause severe environmental problems^3^, negative economic consequences^4^, and impact human health^5^.

**Fig. 1:**
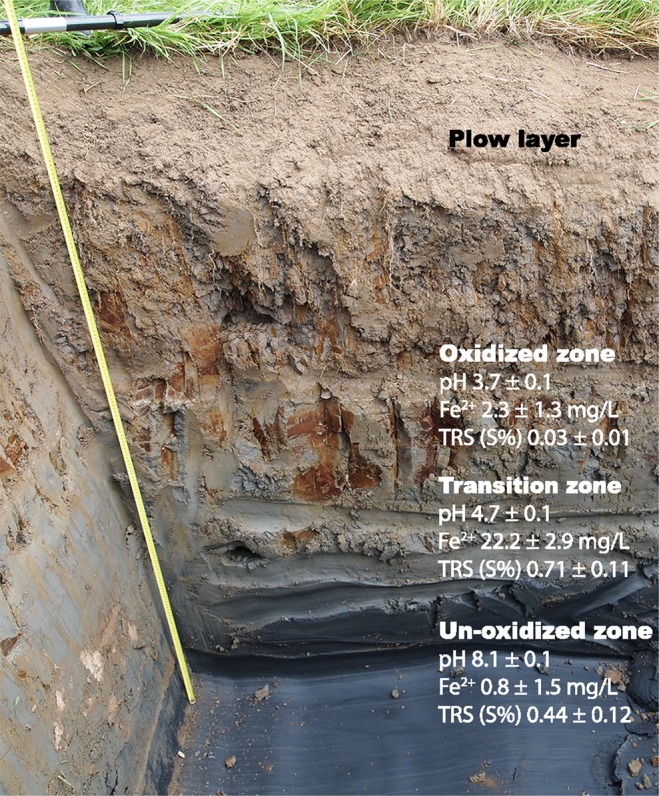
Vertical soil profile of the transition from oxidized ASS to reduced PASS at the Risöfladan experimental field. Basic geochemical information; pH, Fe^2+^, and total reduced sulfur (TRS); is provided for the oxidized (approximately 75–140 cm depth, *n* = 3), transition (approximately 140–190 cm depth, *n* = 3), and un-oxidized soil layers (approximately > 190 cm depth, *n* = 3).

Pyrite oxidation is a complex biogeochemical process that involves a series of chemical reactions aided by microbiological catalysis^1^. Intermediate sulfur species formed during the oxidation process are metabolized by sulfur-oxidizing bacteria^6^ while ferrous iron can be further oxidized at low pH by iron-oxidizing bacteria^7^. Ultimately, pyrite oxidation causes acidification and mobilization of trace metals that are leached to recipient waters. These processes have been extensively studied at the Risöfladan experimental field, Vaasa, Finland (Fig. 1) and there is a large amount of available geochemical and microbial 16S rRNA gene sequencing data^8–11^. The previous 16S rRNA gene based investigations from the oxidized zone at the site identified a mixed community of acidophilic bacteria and archaea similar to that found in acid mine drainage environments along with sulfate reducing bacteria in the underlying un-oxidized potential acid sulfate soil (PASS) zone^10,11^. Finally, 16S rRNA genes with similarity to low temperature adapted microbes were identified in all soil layers, reflecting the boreal environment in Finland. However, more detailed investigations into the metabolic potential and activities of the contrasting microbial communities in oxidized ASS, the transition zone containing the oxidative front, and the PASS have not been reported.

In this Data Descriptor, we present triplicate biological replicate data for 16S rRNA gene amplicons, metagenomes, and metatranscriptomes from the oxidized, transition, and un-oxidized soils horizons at the Risöfladan experimental field (total nine samples) along with the accompanying geochemical metadata (Table 1). The 16S rRNA gene amplicon sequencing dataset contains on average 369 027 reads (min 83 731, max 634 072) for the nine samples (Table 2). In addition, the nine metagenomes (three biological replicates from the three soil types designated OX-MG, TR-MG, and UN-MG) contained 3.78 × 10^9^ paired-end reads (2 × 151 bp) of raw sequence data (Table 3). Finally, the corresponding nine metatranscriptomes (designated OX-MT, TR-MT, and UN-MT) to the three soil types generated 1.11 × 10^11^ paired-end reads (2 × 151 bp) of raw sequence data (Table 3).

**Table 1:** Summary of soil samples and available data.

Sampling zone	Sampling depth (cm below ground)	Replicates	Sample alias	Available data	Ancillary data categories
Oxidized (OX)	75	3	OX-1 to OX-3	16S rRNA gene amplicons, metagenomes, metatranscriptomes	Chemical (pH, EC, redox) and geochemical, Fe- and S- speciation, multi-elements
Transition (TR)	140	3	TR-1 to TR-3	16S rRNA gene amplicons, metagenomes, metatranscriptomes	Chemical (pH, EC, redox) and geochemical, Fe- and S- speciation, multi-elements
Un-oxidized (UN)	190	3	UN-1 to UN-3	16S rRNA gene amplicons, metagenomes, metatranscriptomes	Chemical (pH, EC, redox) and geochemical, Fe- and S- speciation, multi-elements

**Table 2:** Summary of 16S rRNA gene amplicon sequencing of the three soil zones.

Sample	Sequencing Data	Minimum	Mean	Median	Maximum
OX	Raw reads	251 850	318 098	262 356	440 089
TR	Raw reads	83 731	335 070	354 649	566 830
UN	Raw reads	361 455	453 915	366 218	634 072

**Table 3:** Summary of the metagenome (MG) and metatranscriptomes (MT) sequencing of the three soil zones.

Sample	Data	Minimum	Mean	Median	Maximum
OX-MG	Paired-end reads	3.24 × 10^8^	3.70 × 10^8^	3.89 × 10^8^	3.95 × 10^8^
	Total bases	4.90 × 10^10^	5.58 × 10^10^	5.88 × 10^10^	5.97 × 10^10^
TR-MG	Paired-end reads	3.00 × 10^8^	3.82 × 10^8^	3.65 × 108	4.83 × 10^8^
	Total bases	4.53 × 10^10^	5.78 × 10^10^	5.51 × 10^10^	7.29 × 10^10^
UN-MG	Paired-end reads	4.85 × 108	5.06 × 10^8^	5.02 × 10^8^	5.33 × 10^8^
	Total bases	7.32 × 10^10^	7.65 × 10^10^	7.57 × 10^10^	8.04 × 10^10^
OX-MT	Paired-end reads	1.09 × 10^8^	1.43 × 10^8^	1.17 × 10^8^	2.04 × 10^8^
	Total bases	1.65 × 10^10^	2.16 × 10^10^	1.76 × 10^10^	3.08 × 10^10^
TR-MT	Paired-end reads	9.41 × 10^7^	9.73 × 10^7^	9.70 × 10^7^	1.01 × 10^8^
	Total bases	1.42 × 10^10^	1.47 × 10^10^	1.46 × 10^10^	1.52 × 10^10^
UN-MT	Paired-end reads	1.00 × 10^8^	1.31 × 10^8^	1.12 × 10^8^	1.81 × 10^8^
	Total bases	1.51 × 10^10^	1.98 × 10^10^	1.69 × 10^10^	2.73 × 10^10^

The broader goals that motivated the study were to take advantage of multi-omics and geochemical data to gain insights into the metabolic landscape and the molecular mechanisms underlying microbial life in this extreme environment. In addition, based on 16S rRNA gene sequencing many of the microbes in the PASS and ASS are unknown and the data will characterize the structure and function of these microbial populations. Moreover, the microbial metabolic pathways in the actual ASS will be used to answer the question how they catalyze the oxidation process.

The project will be of great use to the research community, as it will generate biological data for which there is hydrological and geochemical monitoring of the site stretching back for many years. Since ASS are extreme environments, the project will be of direct use to researchers, e.g. microbiologists, environmental biologists, geochemists, and geologists. Scientists interested in the iron and sulfur cycles as well as the contaminants released from the ASS during the oxidation process will additionally have a direct use of the project. As sulfidic sediments are affected during dredging, building of infrastructure, and agriculture; researchers involved in these areas will also have use of this work as it may give an explanation in how to minimize the negative impact of these sediments. Finally, the study is relevant to bioinformaticians that study large data sets.

## Methods

### Soil sampling

**Fig. 2:**
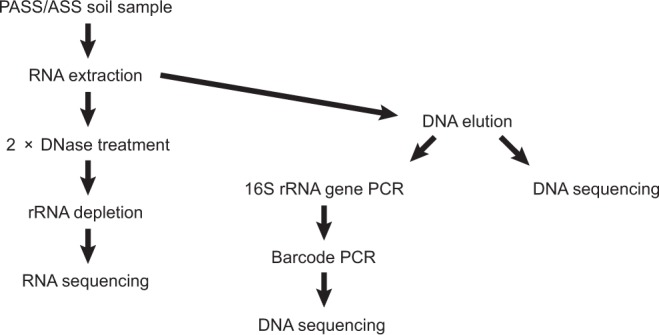
Workflow from potential acid sulfate soil (PASS)/acid sulfate soil (ASS) sampling to deposited sequencing data.

Soil was sampled in mid-August 2017 from the Risöfladan experimental field located in Vaasa, Finland (63° 02′ 50.22″N, 21° 42′ 41.85″E; Figs 1 and 2). Ten replicate soil samples of 5 g were taken from 75 cm below ground (oxidized zone, OX), 140 cm below ground (transition zone, TR), and 190 cm below ground (un-oxidized zone, UN). The samples were placed in separate sterile tubes (30 tubes in total), immediately preserved by freezing in liquid nitrogen, and stored at −80 °C until RNA and DNA were extracted within ten days of sampling.

### RNA and DNA extraction

RNA and DNA were extracted simultaneously from 3 g of soil using the RNeasy® PowerSoil Total RNA Kit (Qiagen) and the following RNeasy® PowerSoil DNA elution Kit (Qiagen). Briefly, 5 g of frozen soil was thawed and carefully mixed before 3 g of soil was transferred to a Bead Tube provided in the RNeasy® PowerSoil Total RNA Kit and RNA was extracted according to the manufacturer’s instructions. The final RNA pellet was suspended in 25 µL RNase/DNase-free water. After eluting the RNA from the RNA Capture Column provided by the RNeasy® PowerSoil Total RNA Kit, the bound DNA was eluted from the RNA Capture Column using the RNeasy® PowerSoil DNA Elution Kit according to the manufacturer’s instructions. The final DNA pellet was suspended in 50 µL RNase/DNase-Free water.

After extraction, RNA samples were treated twice with RNase-free DNase using the Turbo DNA-free™ Kit (Invitrogen) according to the manufacturer’s instructions and then stored at –80 °C. In order to obtain sufficient samples for the final three replicates per soil zone, two to three of the extracted RNA and DNA samples were pooled. The same replicate extractions were pooled for RNA and DNA in order for the metatranscriptomes to match the metagenomes and the RNA transcripts to be mapped to the metagenomes. Ribosomal RNA was depleted from the pooled RNA samples with the Ribominus™ Transcriptome Isolation Kit (Invitrogen) according to the manufacturer’s instructions. Nucleic acid concentrations were measured using a Qubit® 2.0 Fluorometer (Life Technologies). DNA and RNA samples were stored at −80 °C before being submitted to the U.S. Department of Energy Joint Genome Institute (JGI) for sequencing.

### 16S rRNA gene amplicon sequencing

Subsamples from the DNA extracts were used for amplifying the V3-V4 region of the microbial 16S rRNA gene by using primers 341F (CCTACGGGNGGCWGCAG) and 805R (GACTACHVGGGTATCTAATCC)^12^, followed by PCR amplification for Illumina sequencing^13^ (Fig. 2). Samples were sequenced at the Science for Life Laboratory, Sweden, on the Illumina MiSeq platform^14^. A summary of the 16S rRNA gene amplicon sequencing reads is shown in Table 2.

### Metagenome and metatranscriptome library construction and sequencing

For metagenomes, 100 ng of DNA was sheared to 300 bp using the Covaris LE220 and size selected using SPRI beads (Beckman Coulter). The fragments were treated with end-repair, A-tailing, and ligation of Illumina compatible adapters (IDT, Inc) using the KAPA-Illumina library creation kit (KAPA Biosystems). The prepared libraries were quantified using KAPA Biosystem’s next-generation sequencing library qPCR kit and run on a Roche LightCycler 480 real-time PCR instrument. Sequencing of the flowcell was performed on the Illumina NovaSeq sequencer using NovaSeq XP V1 reagent kits, tbd-sample dependant flowcell, following a tbd-sample dependant indexed run recipe.

For metatranscriptomes, stranded cDNA libraries were generated using the Illumina Truseq Stranded RNA LT kit. Ten ng of total RNA was fragmented using divalent cations and high temperature. The fragmented RNA was reversed transcribed using random hexamers and SSII (Invitrogen) followed by second strand synthesis. The fragmented cDNA was treated with end-pair, A-tailing, adapter ligation, and eight cycles of PCR. The prepared libraries were quantified using KAPA Biosystem’s next-generation sequencing library qPCR kit and run on a Roche LightCycler 480 real-time PCR instrument. Sequencing of the flowcell was performed on the Illumina NovaSeq sequencer using NovaSeq XP V1 reagent kits, tbd-sample dependent flowcell, following a tbd-sample dependant indexed run recipe.

The generated reads and bases of all samples can be seen in Table 3.

## Data Records

A summary of the data records included in the study is given in Table 4. The raw Illumina MiSeq 16S rRNA gene sequencing reads are available from the NCBI Sequence Read Archive under the NCBI BioProject accession number PRJNA524144^15^. The raw Illumina NovaSeq sequencing reads for all metagenomes are available from the NCBI Sequence Read Archive under accession numbers SRP185082^16^, SRP185084^17^, SRP185086^18^, SRP185087^19^, SRP185088^20^, SRP185089^21^, SRP185092^22^, SRP185091^23^ and SRP185223^24^. The raw Illumina NovaSeq sequencing reads for all metatranscriptomes are also available from the NCBI Sequence Read Archive under accession numbers SRP185222^25^, SRP185094^26^, SRP185093^27^, SRP185225^28^, SRP185224^29^, SRP185226^30^, SRP185227^31^, SRP185228^32^ and SRP185229^33^.

**Table 4:** Summary of the 16S rRNA gene, metagenome, and metatranscriptome data records.

Sample	NCBI BioProject	NCBI SRA accession	
	16S rRNA	MG	MT
OX-1	PRJNA524144	SRP185082	SRP185222
OX-2	PRJNA524144	SRP185084	SRP185094
OX-3	PRJNA524144	SRP185086	SRP185093
TR-1	PRJNA524144	SRP185087	SRP185225
TR-2	PRJNA524144	SRP185088	SRP185224
TR-3	PRJNA524144	SRP185089	SRP185226
UN-1	PRJNA524144	SRP185092	SRP185227
UN-2	PRJNA524144	SRP185091	SRP185228
UN-3	PRJNA524144	SRP185223	SRP185229

## Technical Validation

Soil samples were taken aseptically by using sterilized equipment and sterile RNase and DNase-free tubes. RNA and DNA were extracted in an RNase free environment and the quantity of the extracted nucleic acids were measured between every step with a Qubit® 2.0 Fluorometer. The quality of the extracted nucleic acids was analyzed using both a NanoDrop™ 2000 Spectrophotometer and agarose gel electrophoresis using a 1% agarose gel with 0.01% SYBR™ Safe DNA Gel Stain (Invitrogen) and a Thermo Scientific™ GeneRuler™ Ready-to-use 1 kb Plus DNA Ladder.

**Fig. 3:**
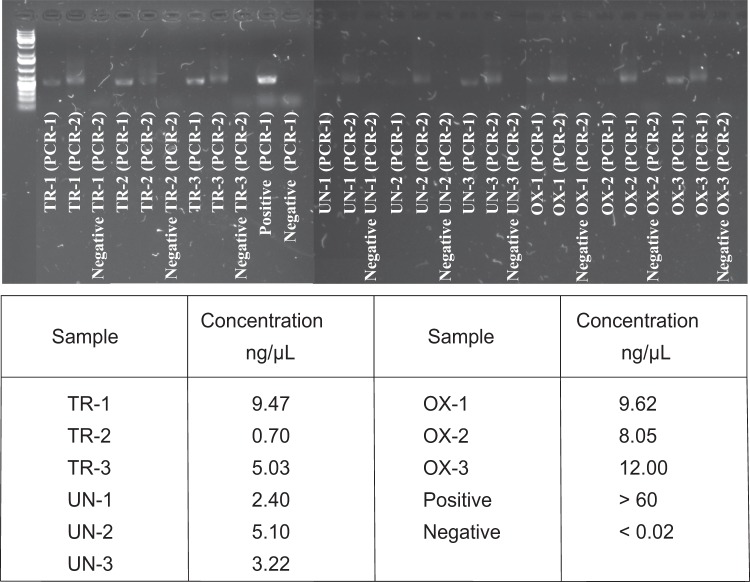
Agarose gel electrophoresis and Qubit® 2.0 Fluorometer DNA concentrations of the V3-V4 region of 16S rRNA gene amplicons from the triplicate samples of the three soil zones. Thermo Scientific™ GeneRuler™ Ready-to-use 1 kb Plus DNA Ladder was used in the gel electrophoresis and can be seen to the left side of the gel. TR-1 to TR-3 are triplicate samples from the transition zone, UN-1 to UN-3 are triplicate samples from the un-oxidized zone, and OX-1 to OX-3 are triplicate samples from the oxidized zone. PCR-1 stands for templates after the first amplification step by using primers 341F and 805R. The positive (DNA from *Pseudoateromonas citrea*) and negative (RNase/DNase-free water) controls used in the first amplification are named “Positive” and “Negative” in the figure. PCR-2 stands for templates after the second amplification step that attaches the barcodes identifying the sequences in the MiSeq Illumina sequencing. Negative PCR-2 stands for negative controls in the second amplifications step, i.e. mastermix with primer-barcodes without templates.

For 16S rRNA gene amplification, negative and positive controls in the form of RNase/DNase-free water and DNA extracted from *Pseudoateromonas citrea*, respectively were included in the first amplification step. In addition, negative controls in the form of mastermix with primer-barcodes without templates were included in the second PCR amplification to confirm that both the primers and the barcodes identifying the sequences functioned correctly and that no contamination occurred. The concentration of the 16S rRNA gene amplicons and controls was measured with a Qubit® 2.0 Fluorometer and their quality were analyzed using agarose gel electrophoresis (Fig. 3).
